# Multilocular Ovarian Cystic Tumor—Operation—Recovery

**Published:** 1893-03

**Authors:** F. B. Magruder

**Affiliations:** San Angelo, Texas


For Daniel's Texas Medical Journal.

### MULTILOCULAR OVARIAN CYSTIC TUMOR—OPERATION—RECOVERY.

BY F. B. MAGRUDER, M. D., SAN ANGELO, TEXAS.

ON December 13, 1892, I removed from Mrs. K., of San Angelo, Texas, a large multilocular cystic ovarian tumor, weighing twenty pounds.

Mrs. K. is fifty-five years old, the mother of seven children; for the last few years has been in rather poor health, having suffered a great deal from chronic cystitis.

About twelve months ago she discovered a tumor, in the region of the right ovary, about the size of an orange. It gave her a great deal of pain for some two months, which gradually subsided without treatment, but continued to grow rapidly, until it obtained the above dimensions and she could hardly get about, with great difficulty getting up when she was down.

She was very much alarmed about her condition.

I was called to see her December 5; diagnosed multilocular cystic ovarian tumor, and advised its removal at once, which she readily consented to.

One week's treatment put her in very good condition for an operation. On December 13, assisted by Drs. Wm. Perrin, L. S.

Smith and M. Mays, chloroform was administered, I made abdominal incision about three inches in the median line.

The first cyst opened contained colored water; second cyst contained a thick jelly-like fluid that would not discharge through a large trocar; third cyst contained a dark jelly-like substance, and so on. No two contained the same kind of liquid.

I enlarged the opening to five inches and drew the tumor out, there being no adhesions; the pedicle was about four inches broad.

It was secured by braided silk in two sections; tumor removed and stump dropped in the cavity. (The left ovary was carefully examined and found to be normal.) The peritoneum closed separately with fine cat-gut, the abdomen closed with silk-worm gut, sprinkled with iodoform and covered with iodoform gauze. All covered with absorbent cotton and a roller bandage applied. *No drainage was used.* Within one hour from the time we commenced giving chloroform the patient was snugly in bed and had rallied from the effects of the anesthetic and was feeling comfortable.

The first twenty-four hours the temperature rose to 100° and remained so for three days, when it rose to 101°, but soon decreased without treatment to 99°, where it remained for eight days.

I attribute the rise of fever to the condition of the bladder, as it was very irritable and contained a good deal of bloody mucus. The bladder was washed out every day with a solution of boracic acid and finally with a solution of nitrate of silver. On the tenth day all irritability had subsided, she passed the urine without discomfort and the temperature dropped to normal. On the tenth day the stitches were removed and the wound found to be entirely healed. Not one drop of pus or discharge of any kind was ever detected in or about the wound. On the twelfth day she set up in bed, and in due time convalescence was established. At this time, five weeks after the operation, she is able to leave the house.

I report this case not because of anything new, but for the encouragement of some of our medical brethren who think laparotomy should not be performed outside of our large city hospitals, and then only by such as make a specialty of abdominal surgery,—and to put on record one more successful case.

